# A combination of biomarkers for predicting stallion sperm fertility

**DOI:** 10.1007/s11259-024-10372-6

**Published:** 2024-04-23

**Authors:** Anders Johannisson, Jane M. Morrell, Theodoros Ntallaris

**Affiliations:** 1 Clinical Sciences, Swedish University of Agricultural Sciences (SLU), Uppsala, Sweden; 2 Present Address: Department of Medical Sciences, Uppsala University, Uppsala, Sweden

**Keywords:** Flow-cytometric analysis, Sperm kinematics, Artificial insemination

## Abstract

Equine breeding would benefit greatly from reliable biomarkers of stallion or ejaculate fertility. The aim of the study was to investigate how several in vitro sperm characteristics correlate with fertility after artificial insemination, to explore the potential to build a fertility prediction model for stallions. Cooled insemination doses (3–5 per stallion) were obtained from various studs. Sperm membrane integrity, acrosome integrity, chromatin integrity, mitochondrial membrane potential, and reactive oxygen species production were evaluated by flow cytometry 24–30 h after semen collection, and sperm motility was assessed by computer aided sperm analysis. Calcein violet was used to differentiate viable spermatozoa. Per season pregnancy rates for these stallions were available the following year. Positive correlations were found between pregnancy rate and straightness (r = 0.43, p ≤ 0.001), as well as pregnancy rate and the proportion of living hydrogen peroxide positive spermatozoa (r = 0.32, p ≤ 0.05). There were negative correlations between pregnancy rate and amplitude of lateral head displacement (r = -0.26, p ≤ 0.05), and between pregnancy rate and the mean fluorescence of dead superoxide positive spermatozoa (r = -0.46, p < 0.001). Principal component analysis indicated that motility, membrane integrity, DNA fragmentation, and reactive oxygen species production were associated with pregnancy rate. Therefore, a combination of these factors could be used as a biomarker of fertility when assessing ejaculates. However, data from more individuals would be required to construct a model for fertility prediction.

**Supplementary Information:**

The online version contains supplementary material available at 10.1007/s11259-024-10372-6.

## Introduction

Although reliable indicators for predicting the likely fertility of stallions is desired by many equine breeders, identifying such biomarkers is challenging. Not only are individuals (stallions and mares) selected for breeding based on physical or athletic attributes that have little to do with fertility, but the number of mares covered or bred by artificial insemination (AI) by one stallion in a season is low compared to livestock species (Griffin et al. 2019). Furthermore, there is no single “standard” protocol regarding the number of sperm required for artificial insemination (Malaluang et al. 2024), making it difficult to compare pregnancy rates between different countries or between different studs.

Seasonal pregnancy rates after AI with cooled semen are generally around 65–68% (Rota et al 2004), although some stallions achieve much lower rates (Egyptien et al. 2023). It would be useful to have a reliable means of identifying stallions of low fertility, or even specific ejaculates that are substandard, without the necessity for inseminating mares and waiting several weeks for a preliminary result. Semen quality evaluation at the stud is usually restricted to a subjective assessment of sperm motility immediately after semen collection (Malmgren 1994), despite the fact that measurement of subjective motility is not generally indicative of sperm fertilizing ability (Watson 1990; Morrell et al 2017).

Sperm quality can be evaluated in the laboratory by a combination of tests for structural and functional parameters such as morphology, motility, plasma membrane integrity, acrosomal membrane integrity, mitochondrial function, chromatin structure, capacitation and fertilization (Graham and Moce 2005). This is done by visual analysis of unstained and stained spermatozoa, or by automated sperm analyses using computer assisted sperm analysis (CASA), flow cytometry and, in some species, in vitro fertilization (IVF) (Morrell and Rodriguez-Martinez 2011; Graham and Moce 2005). Apart from IVF, these assays represent attempts to evaluate various aspects of sperm function as an indirect indicator of sperm fertilizing potential (Bollwein & Malama 2023), but are limited by their lack of ability to evaluate more than one function. This limitation can be overcome to some extent by combining the results of different independent assays, for example assays for sperm DNA integrity and calcium ion concentration (Wach-Gygax et al. 2017). However, the potential of enhancing fertility predictions for stallions by combining different sperm assays has not been fully explored.

Studies of the prediction of bull fertility within our group (Kumaresan et al 2017) have yielded a successful model for discriminating between above- and below-average fertility bulls, based on in vitro tests. The combined model developed for bulls incorporated results from sperm viability, chromatin integrity and hydrogen peroxide status, and achieved a high degree of accuracy (r^2^ = 0.83). The individual tests, while still achieving significant prediction, yielded lower values. It was previously shown that additional information regarding sperm quality of stallions can be obtained by a simultaneous staining for mitochondrial membrane potential and superoxide (Johannisson et al 2018). Recent work (Al-Kass et al 2023) showed that various properties of stallion sperm chromatin have a correlation with fertility. Other researchers have identified different variables of sperm quality as potential biomarkers, e.g. Albrizio et al. (2018) considered that mitochondrial membrane potential was a good biomarker of fertility.

The aim of the present study was to investigate how several in vitro sperm characteristics correlate with each other, and also with fertility, and to explore if it is possible to build a fertility prediction model from these parameters.

## Material and Methods

### Semen collection

Commercial semen doses were obtained from 14 Warmblood stallions, trotters and sport horses, 4–18 years old, kept on commercial studs in Sweden. From each stallion 3–5 semen samples were obtained during February-August, giving a total of 60 ejaculates analysed.

### Sperm analysis

On each occasion, AI doses of one billion motile spermatozoa (the standard dose for cooled semen in Sweden) were aspirated into 20-mL syringes. The semen doses were transported to other stud farms and to the laboratory at the Swedish University of Agricultural Sciences (SLU) in a Styrofoam box containing a cold pack; such packaging maintains the temperature of semen doses at approximately 7ºC for 24 h when the ambient temperature is 20ºC (Malmgren, 1998). At SLU, the sperm concentration was measured using a Nucleocounter SP-100 (Chemometec, Allerød, Denmark) to establish the sperm concentration for staining the spermatozoa for flow cytometry, which was performed at 24 h after semen collection.

### Computer-aided sperm analysis (CASA)

Motility analysis (CASA) was performed using a SpermVision (Minitüb, Tiefenbach, Germany), connected to an Olympus BX 51 microscope (Olympus, Japan) with a warm stage at 38ºC, when the samples arrived and again after 24 h. Aliquots (6 μL) of sperm samples were pipetted on to a warm glass slide and covered with an 18 × 18-mm coverslip. Motility and kinematics in eight fields (~ 1000 spermatozoa) were evaluated using the SpermVision software program. Cell identification area was set at 14–80 µm^2^; spermatozoa were classified as immotile spermatozoa if the average change in the orientation of the head was less than 17º; and local (i.e. non-progressive) motile spermatozoa were those covering a straight line distance (DSL) < 6 µm, or having a circular movement with a radius < 35 µm and DSL < 30 µm. Apart from evaluation of total motility and progressive motility (%), the kinematics measured were curvilinear velocity (VCL, µm/s), straight line velocity (VSL, µm/s), average path velocity (VAP, µm/s), straightness (STR), linearity (LIN), Wobble (WOB), amplitude of lateral head deviation (ALH µm) and beat cross frequency (BCF, Hz).

### Chromatin integrity

The method used for the measurement of chromatin integrity was based on previously published methods (Johannisson et al. 2009) with minor modifications. Equal volumes (50 µL) of sperm samples and buffer containing 0.01 M Tris–HCl, 0.15 M sodium chloride and 1 mM EDTA (pH 7.4; Tris-sodium-EDTA buffer, TNE) were mixed to give a final sperm suspension of approximately 2 × 10^6^ cells mL-1; samples were snap-frozen in liquid nitrogen before being transferred to a -80ºC freezer for storage until subsequent evaluation by flow cytometry. Samples were thawed on crushed ice immediately before staining: 90 µl of TNE-buffer was added to 10 µl of each thawed sample. The TNE-extended sperm suspensions were mixed with 0.2 mL of a low-pH detergent solution containing 0.17% Triton X-100, 0.15 M NaCl and 0.08 M HCl (pH 1.2) for partial DNA denaturation in situ, and were then stained 30 s later with 0.6 mL acridine orange (AO) (6 µg mL-1 in 0.1 M citric acid, 0.2 M Na_2_HPO4, 1 mM EDTA, 0.15 M NaCl, pH 6.0). Measurements were made with a FACSVerse flow cytometer (BDBiosciences, San José, CA, USA) equipped with standard optics. From each sample, a total of 10 000 events was evaluated at a flow rate of approximately 200 cells s-1 after illumination with a blue laser (488 nm). Green fluorescence from AO bound to double-stranded DNA was detected through a 527/32 bandpass filter, whereas red fluorescence from AO bound to single-stranded DNA was detected through a 700/54 bandpass filter. Further calculations of DNA fragmentation index (%DFI) as well as enumeration of spermatozoa with High DNA Stainability (%HDS) were performed using FCSExpress version 5 (DeNovo Software, Thornthill, Ontario, Canada).

### Simultaneous measurement of mitochondrial membrane potential and ROS

Measurements of MMP were made by staining spermatozoa with the lipophilic substance 5,5,6,6-tetrachloro-1,1,3,3-tetraethylbenzimidazolyl carbocyanine iodide (JC-1). This metachromatic dye differentially labels mitochondrial activity according to membrane potential, emitting in the high orange wavelength for high MMP and in the green wavelength for low MMP, when excited at 488 nm. Measurements of ROS were made by staining spermatozoa with MitoSOXRed (Invitrogen, Carlsbad, CA, USA). MitoSOXRed is a specific fluorescent probe for SO^□^ produced by mitochondria in the cell population. The samples were also stained with Calcein Violet-AM (Invitrogen) to restrict analysis to spermatozoa with esterase activity at a final concentration of 0.3 µM; menadione, a stimulant of ROS production, was also added. A cell suspension with 2 million sperm/mL (final volume of 300 µL) was stained. The samples were divided in two groups: the first one was incubated with a final concentration of 1.5 µM of JC-1, 3 µM of MitoSOXRed and 0.3 µM Calcein Violet; the second group was incubated with a final concentration of 1.5 µM of JC-1, 3 µM of MitoSOXRed, 0.3 µM Calcein Violet and 200 µM menadione.

After incubation for 30 min at 37ºC, the samples were analysed using a FACSVerse flow cytometer. Samples were excited with a blue laser (488 nm) and a violet laser (405 nm). Green fluorescence was detected with a bandpass filter (527/32 nm), orange fluorescence was detected using a bandpass filter (586/42 nm), red fluorescence was measured using a bandpass filter (700/54 nm) and blue fluorescence was detected with a 528/45 nm bandpass filter. The data were compensated to eliminate spectral overlap.

A total of 30 000 events was evaluated and calculated as percentages of spermatozoa with high or low mitochondrial membrane potential, superoxide negative and live or superoxide positive, after gating for sperm cells in the forward scatter (FSC)-side scatter (SSC) dot-plot and also excluding spermatozoa negative for Calcein Violet. Cells were classified as having either high or low MMP and high or low ROS production by placing a quadrant based on the menadione-stimulated sample as indicated in our previous paper (Johannisson et al 2018).

### Superoxide and hydrogen peroxide

The method was modified from Johannisson et al. (2014), the modification being that Calcein Violet was used instead of Hoechst 33258 to differentiate viable cells. Semen (300 µl) was stained with Calcein Violet-AM (final dye concentration 0.3 µM), hydroethidine (HE) (final dye concentration 0.4 µM) and dihydrodichlorofluorodiacetate H_2_DCFDA (final dye concentration 20 µM), purchased from Invitrogen Molecular Probes. After a 30-min incubation, the samples were analysed with a FACSVerse flow cytometer, excitation with an blue laser (488 nm) and a violet laser (405 nm). Green fluorescence was detected with a bandpass filter (527/32 nm), orange fluorescence was detected using a bandpass filter (586/42 nm), red fluorescence was measured using a bandpass filter (700/54 nm) and blue fluorescence was detected with a 528/45 nm bandpass filter. After gating for sperm cells in the forward scatter-side scatter dotplot, 30 000 sperm were evaluated and characterised as living superoxide negative, living superoxide positive and dead superoxide in the HE dotplot, living hydrogen peroxide negative, living hydrogen peroxide positive, dead hydrogen peroxide negative and dead hydrogen peroxide positive in the H_2_DCFDA dotplot.

### Membrane integrity

Membrane integrity (MI) was analysed after staining with SYBR-14 and propidium iodide (PI; Live-Dead Sperm Viability Kit L-7011; Invitrogen, Eugene, OR, USA). Aliquots were adjusted to a concentration of 2 million sperm/ml with CellWash (final volume of 300 µL) and were stained with 0.6 µL SYBR-14 stock solution (diluted 1: 50 in CellWash) and 3.0 µL PI. After incubating for 10 min at 37ºC, spermatozoa were analysed using a FACSVerse Flow Cytometer with standard optics. In total, 30 000 events were collected and quantified as percentages of sperm populations. Samples were excited with a blue laser (488 nm). Green fluorescence was detected with an FL1 bandpass filter (527/32 nm), whereas red fluorescence was measured using an FL3 bandpass filter (700/54 nm). The spermatozoa were classified as live spermatozoa with an intact membrane (SYBR + -14/PI-), moribund (SYBR–14/PI +), and dead (SYBR + -14/PI +).

### Mitochondrial membrane potential

For evaluation of the mitochondrial potential without correlation to ROS production, 1.2 μL JC-1 (stock 900 µM) was mixed with 300 μL sperm sample in Cellwash to a final concentration of 3.6 µM and incubated for at least 30 min at 37 ^◦^C before analysis. JC-1 fluorescence was measured in the FL1 (527/32 nm) and FL2 (586/42 nm) channels of the FACSVerse flow cytometer. A total number of 30,000 cells was evaluated and classified as percentages in two distinct groups: sperm cells with high respiratory activity emitting orange fluorescence, and low respiratory activity emitting green fluorescence.

### Acrosome integrity

For evaluation of acrosome integrity, a combination of FITC-PNA to evaluate acrosome reaction, PI to evaluate membrane integrity and Hoechst 33,342 to exclude debris. Sperm samples in Cellwash at a concentration of 2*10^6^ cells/ml were stained with final concentrations of Hoechst 33,342 10 µg/ml (Sigma-Aldrich), 24 µM PI (Invitrogen, from kit L-7011) and 5 µg/ml FITC-PNA (Sigma-Aldrich). *After incubation at 37ºC for 30 min*, samples were excited with a blue laser (488 nm) and a violet laser (405 nm). Green fluorescence from FITC-PNA was detected with a bandpass filter (527/32 nm), red fluorescence from PI was measured using a bandpass filter (700/54 nm) and blue fluorescence from Hoechst 33,342 was detected with a 528/45 nm bandpass filter. The data were compensated to eliminate spectral overlap. After gating for spermatozoa using FSC and SSC as well as elimination of debris using Hoechst 33,342, spermatozoa were classified into four groups, live acrosome-reacted, dead acrosome-reacted, live non-reacted and dead non-reacted.

## Fertility

Pregnancy rate per season was calculated for the sampling year, excluding stallions with fewer than 10 inseminated mares. Pregnancy rate was calculated as the number of pregnant mares/number of inseminated mares * 100. The number of inseminated mares per stallion ranged from 15 to 320.

## Statistical analysis

Statistical analyses were conducted using SAS® software (version 9.4; SAS Institute Inc., Cary, NC). Data were tested for homogeneity of variance by Bartlett's test and for normal distribution by the Shapiro Wilk test. Descriptive statistics (mean, standard deviation, median, boxplots) were calculated using the MEANS and SGPLOT procedure in the software. Stallion fertility was arbitrarily classified as above or below average using 70% as the threshold. This threshold was used to calculate the sensitivity and specificity using the FREQ procedure in SAS software. Data on sperm properties were analyzed using PROC MIXED in the SAS software. The data for live H_2_O_2_^+^ and for low MMP and high SO deviated from a normal distribution and were log transformed. However, to improve clarity, avoid redundancy and facilitate interpretation, the untransformed values are presented throughout.

Least squares means (LSM ± SEM) estimated by the models were adjusted using the Scheffé adjustment for multiple post-ANOVA comparisons and compared.

The CORR procedure was used to compute correlation coefficients for the different sperm properties. To group the parameters with similar trends, the 37 sperm functional attributes measured were subjected to principal component analysis (PCA) using the Varimax method. The parameters were grouped in two components as per rotated component matrix, and were placed in the group where the highest positive value was observed.

The alpha value for this experiment was selected as 5%. Differences between 0.05 < p ≤ 0.10 were considered trends.

## Results

**Fig. 1:**
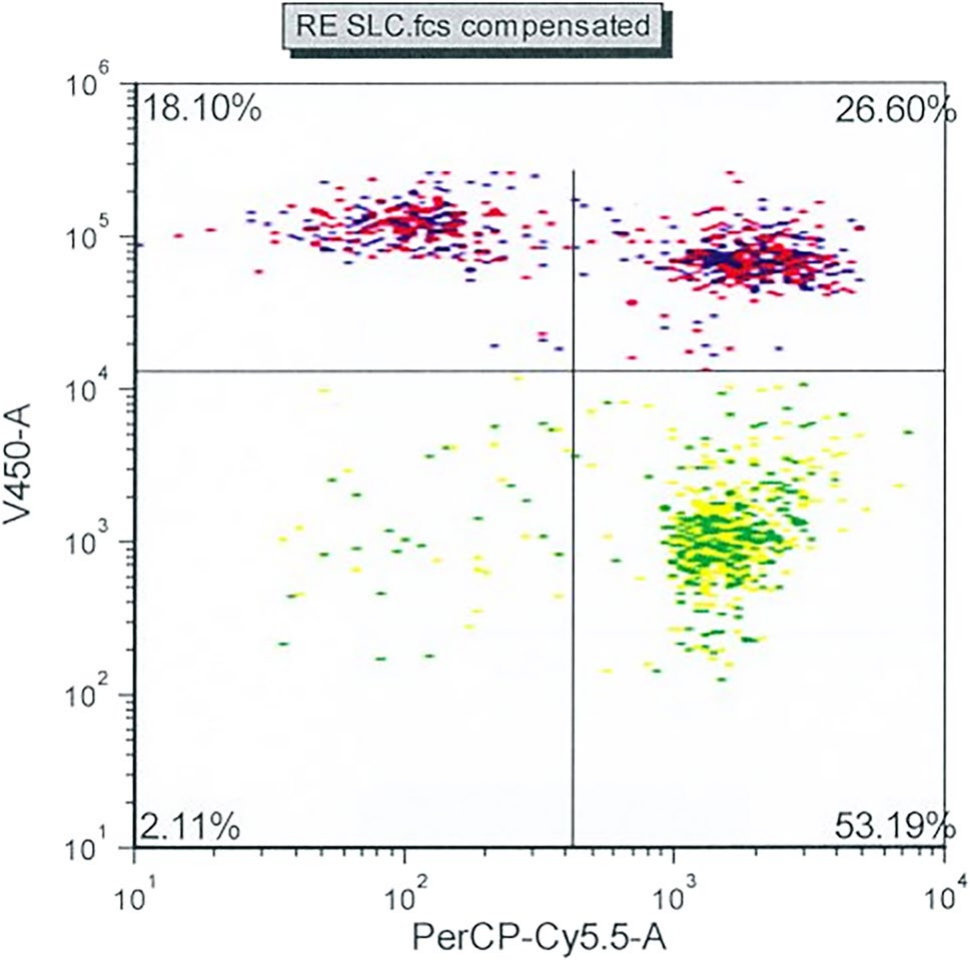
Dot-plot of fluorescence from Calcein Violet and Hydroethidine, for evaluation of superoxide production and viability. The following cell populations are seen: Upper left, live superoxide negative; upper right, live superoxide positive; lower left, dead superoxide negative; lower right, dead superoxide positive

A dot-plot of the fluorescence from sperm aliquots stained with calcein violet and hydroethidine is shown in Fig. 1. Four sub-populations could be identified, corresponding to live, superoxide negative spermatozoa; live, superoxide positive spermatozoa; dead, superoxide negative spermatozoa; and dead, superoxide positive spermatozoa.

The mean values for all stallions and parameters are given in Tables 1, 2, 3, 4 and 5

**Table 1:** Stallion sperm kinematics (mean ± SD) (n = 14 stallios, 60 ejaculates)

Stallion (no. ejaculates)	TM(%)	PM (%)	VAP (µm/s)	VCL (µm/s)	VSL (µm/s)	STR	LIN	WOB	ALH (µm)	BCF Hz
1 (3)	77.00 ± 1.00	46.33 ± 1.15	79.33 ± 4.73	136.33 ± 11.72	55.33 ± 2.89	0.69 ± 0.01	0.40 ± 0.02	0.58 ± 0.01	4.49 ± 0.07	29.64 ± 0.58
2 (3)	73.00 ± 10.54	48.33 ± 8.02	80.67 ± 0.58	146.67 ± 5.13	57.67 ± 0.58	0.71 ± 0.01	0.39 ± 0.01	0.54 ± 0.02	4.33 ± 0.08	30.04 ± 0.34
3 (5)	48.00 ± 7.91	33.00 ± 5.61	68.40 ± 8.26	109.20 ± 12.79	55.80 ± 8.44	0.81 ± 0.03	0.51 ± 0.03	0.62 ± 0.02	3.47 ± 0.45	34.00 ± 4.16
4 (9)	68.67 ± 13.56	43.56 ± 11.83	81.89 ± 3.44	140.11 ± 9.66	60.44 ± 3.05	0.73 ± 0.03	0.45 ± 0.08	0.58 ± 0.03	4.18 ± 0.35	30.82 ± 1.64
5 (5)	39.60 ± 5.94	26.80 ± 5.63	71.80 ± 5.54	126.40 ± 12.12	56.00 ± 3.24	0.78 ± 0.02	0.44 ± 0.03	0.56 ± 0.03	3.85 ± 0.20	29.96 ± 1.35
6 (3)	75.33 ± 3.21	55.67 ± 4.51	94.67 ± 9.61	164.67 ± 19.66	71.00 ± 7.55	0.75 ± 0.02	0.43 ± 0.02	0.57 ± 0.01	4.44 ± 0.31	32.27 ± 2.21
7 (4)	62.25 ± 26.47	42.50 ± 22.58	54.00 ± 10.42	91.50 ± 18.91	40.75 ± 9.95	0.75 ± 0.06	0.44 ± 0.05	0.59 ± 0.03	3.16 ± 0.14	27.48 ± 5.98
8 (4)	87.75 ± 6.9	61.75 4.99	79.50 ± 3.79	138.00 ± 6.98	59.75 ± 3.10	0.75 ± 0.03	0.43 ± 0.02	0.55 ± 0.05	4.23 ± 0.32	32.87 ± 2.24
9 (4)	64.00 ± 20.69	45.50 ± 12.97	75.25 ± 10.50	134.25 ± 20.07	57.25 ± 8.26	0.76 ± 0.01	0.42 ± 0.03	0.55 ± 0.04	3.85 ± 0.15	33.93 ± 3.93
10 (5)	69.40 ± 17.40	46.80 ± 14.89	77.80 ± 5.36	133.20 ± 11.12	58.20 ± 4.60	0.74 ± 0.01	0.43 ± 0.01	0.58 ± 0.02	4.35 ± 0.33	30.63 ± 2.05
11 (4)	78.75 ± 6.85	57.25 ± 3.30	69.00 ± 4.55	122.50 ± 2.89	53.75 ± 2.63	0.78 ± 0.01	0.44 ± 0.01	0.57 ± 0.00	3.60 ± 0.39	32.81 ± 0.81
12 (4)	67.00 ± 11.11	48.25 ± 7.41	65.75 ± 4.35	116.25 ± 10.24	51.00 ± 3.83	0.77 ± 0.02	0.44 ± 0.03	0.56 ± 0.03	3.72 ± 0.19	30.30 ± 0.88
13 (3)	70.67 ± 7.51	56.33 ± 9.81	69.00 ± 23.64	118.33 ± 40.51	55.67 ± 23.59	0.79 ± 0.07	0.46 ± 0.05	0.58 ± 0.03	3.43 ± 0.80	30.99 ± 1.99
14 (4)	70.25 ± 4.72	50.25 ± 6.75	82.50 ± 8.10	140.50 ± 13.03	65.25 ± 8.62	0.79 ± 0.03	0.46 ± 0.03	0.58 ± 0.02	4.16 ± 0.28	31.95 ± 1.22

*TM*, total motility; *PM*, progressive motility; *VCL*, curvilinear velocity; *VSL*, straight line velocity; *AVP*, average path velocity; *STR*, straightness; *LIN*, linearity; *WOB*, wobble; *ALH*, amplitude of lateral head displacement; *BCF*, beat cross frequency

**Table 2:** Stallion sperm characteristics (membrane integrity, acrosome status, chroamtin integrity and high staining DNA, together with per season pregnancy rate (n = 14 stallions, 60 ejaculates)

Stallion (no. ejaculates)	Pregnancy rate (%)	Membrane intact (%)	Dead non-reacted	Dead acrosome-reacted	Live non-reacted	Live acrosome reacted	JC + (%)	%DFI	Higreen (%)
1 (3)	20.00	70.03 ± 8.98	11.45 ± 0.23	23.12 ± 12.82	65.15 ± 12.88	0.29 ± 0.17	52.98 ± 10.12	24.70 ± 9.70	0.44 ± 0.23
2 (3)	30.00	66.06 ± 11.73	13.65 ± 5.19	15.97 ± 1.57	69.67 ± 7.04	0.72 ± 0.29	56.79 ± 2.06	31.30 ± 6.74	0.50 ± 0.25
3 (5)	57.00	67.49 ± 5.75	18.68 ± 3.85	17.57 ± 5.83	63.28 ± 8.26	0.46 ± 0.23	64.16 ± 9.26	32.04 ± 409	0.77 ± 0.30
4 (9)	62.00	72.85 ± 8.29	12.13 ± 5.20	20.72 ± 6.26	66.59 ± 10.98	0.56 ± 0.30	60.76 ± 12.30	22.45 ± 8.29	0.28 ± 0.21
5 (5)	70.00	62.10 ± 4.44	14.77 ± 2.71	33.43 ± 10.90	51.08 ± 13.08	0.72 ± 0.30	59.96 ± 17.69	25.98 ± 4.05	0.53 ± 0.36
6 (3)	73.00	75.80 ± 7.33	8.48 ± 1.57	19.75 ± 6.39	70.60 ± 8.07	1.18 ± 0.23	67.30 ± 9.58	17.28 ± 2.55	0.21 ± 0.13
7 (4)	77.00	59.87 ± 15.65	20.24 ± 6.85	22.08 ± 14.10	56.97 ± 16.32	0.72 ± 0.20	44.04 ± 28.21	25.11 ± 5.97	0.15 ± 0.08
8 (4)	79.00	77.81 ± 20.20	19.17 ± 18.56	9.95 ± 2.37	70.18 ± 18.00	0.20 ± 0.05	38.93 ± 28.58	9.85 ± 2.87	0.29 ± 0.08
9 (4)	79.00	65.48 ± 14.25	21.61 ± 4.11	14.10 ± 12.31	63.91 ± 15.06	0.38 ± 0.02	38.59 ± 17.18	28.54 ± 10.98	0.53 ± 0.06
10 (5)	79.00	41.17 ± 10.87	33.09 ± 5.96	29.63 ± 10.83	36.98 ± 12.88	0.29 ± 0.07	44.37 ± 27.16	47.39 ± 11.19	0.28 ± 0.12
11 (4)	83.00	69.76 ± 13.29	23.80 ± 11.22	15.18 ± 2.48	60.27 ± 8.58	0.75 ± 0.62	32.68 ± 19.66	17.61 ± 1.84	0.32 ± 0.17
12 (4)	83.00	77.60 ± 5.60	14.54 ± 1.10	10.23 ± 5.89	75.01 ± 6.73	0.22 ± 0.15	66.79 ± 9.21	18.46 ± 3.33	0.74 ± 0.13
13 (3)	85.00	84.17 ± 3.72	5.58 ± 1.14	12.02 ± 4.65	82.15 ± 5.93	0.26 ± 0.16	57.28 ± 2.89	15.23 ± 1.16	0.05 ± 0.03
14 (4)	93.00	83.44 ± 4.32	6.73 ± 1.59	12.52 ± 3.49	80.54 ± 4.81	0.21 ± 0.05	77.73 ± 4.62	16.67	0.35

*%DFI*, DNA fragmentation index; *JC*, high mitochondrial membrane potential; *HiGreen*, high DNA staining spermatozoa

**Table 3:** Stallion sperm characteristics and per season pregnancy rate mitochondrial membrane potential and mitochondrial superoxide production (n = 14 stallions, 58 ejaculates)

Stallion (ejac no.)	Pregnancy rate (%)	% Live	low MMP high SO (%)	high MMP high SO (%)	low MMP low SO (%)	High MMP low SO (%)
1 (3)	20.00	59.58 ± 10.39	5.34 ± 0.37	2.65 ± 1.61	5.94 ± 6.39	86.09 ± 8.37
2 (1)	30.00	70.78	8.10	2.39	0.13	89.37
3 (5)	57.00	67.11 ± 6.55	14.63 ± 5.93	25.20 ± 19.89	1.97 ± 1.41	58.16 ± 24.59
4 (9)	62.00	58.17 ± 14.14	9.48 ± 10.13	15.43 ± 13.22	2.55 ± 1.95	72.54 ± 23.68
5 (5)	70.00	44.83 ± 10.79	12.50 ± 13.69	23.56 ± 21.61	0.96 ± 1.24	62.95 ± 30.57
6 (3)	73.00	59.83 ± 9.29	3.50 ± 1.02	10.17 ± 3.02	0.59 ± 0.86	85.48 ± 1.00
7 (4)	77.00	57.91 ± 12.22	48.58 ± 25.77	4.21 ± 1.23	5.74 ± 3.00	41.41 ± 29.00
8 (4)	79.00	80.67 ± 4.00	33.81 ± 33.82	8.10 ± 6.76	6.42 ± 3.63	51.68 ± 34.81
9 (4)	79.00	64.95 ± 16.61	29.23 ± 14.58	14.78 ± 21.02	5.17 ± 3.73	50.83 ± 26.70
10 (5)	79.00	36.33 ± 12.95	30.46 ± 25.44	5.81 ± 3.61	5.41 ± 6.23	58.30 ± 30.43
11 (4)	83.00	67.42 ± 3.93	36.10 ± 14.37	13.14 ± 11.33	16.22 ± 6.12	34.55 ± 15.48
12 (4)	83.00	73.60 ± 7.98	14.45 ± 11.08	8.82 ± 14.45	9.53 ± 9.72	67.19 ± 23.98
13 (3)	85.00	69.63 ± 5.61	24.76 ± 14.62	9.63 ± 7.77	7.99 ± 8.64	57.45 ± 10.67
14 (4)	93.00	74.69 ± 4.06	4.05 ± 2.00	9.23 ± 2.92	0.27 ± 0.04	86.43 ± 3.19

*MMP*, mitochondrial membrane potential; *SO*, superoxide

**Table 4:** Stallion sperm hydrogen peroxide status (n = 14 stallions; 58 ejaculates)

Stallion (ejac no.)	Live H2O2 Negative	Live H2O2 Positive	Dead H2O2 Negative	Dead H2O2 positive	MFI Live H2O2 positive	MFI Dead H2O2 positive
1 (3)	63.18 ± 11.49	0.23 ± 0.01	36.38 ± 11.45	0.23 ± 0.05	41.11 ± 6.05	52.80 ± 12.77
2 (1)	72.38	0.10	27.45	0.06	27.22	32.16
3 (5)	70.00 ± 4.61	0.31 ± 0.25	29.18 ± 4.73	0.51 ± 0.56	32.88 ± 11.86	39.32 ± 17.52
4 (9)	66.27 ± 8.44	0.52 ± 0.26	32.99 ± 8.11	0.22 ± 0.18	38.74 ± 9.60	44.76 ± 12.75
5 (5)	52.55 ± 12.15	0.31 ± 0.13	46.38 ± 12.23	0.76 ± 0.89	37.28 ± 7.02	44.97 ± 7.88
6 (3)	61.73 ± 10.15	0.79 ± 0.62	36.77 ± 11.08	0.70 ± 0.55	32.90 ± 0.70	42.02 ± 2.02
7 (4)	66.28 ± 13.58	0.74 ± 0.65	32.86 ± 14.02	0.12 ± 0.08	26.60 ± 7.49	26.93 ± 8.80
8 (4)	81.09 ± 3.33	0.58 ± 0.29	18.26 ± 3.61	0.09 ± 0.12	30.68 ± 3.08	33.49 ± 4.71
9 (4)	65.39 ± 17.72	0.26 ± 0.05	34.04 ± 17.47	0.32 ± 0.24	32.16 ± 0.12	35.70 ± 11.36
10 (5)	38.79 ± 9.86	0.49 ± 0.44	59.92 ± 8.88	0.80 ± 0.84	37.39 ± 14.39	40.49 ± 14.08
11 (4)	67.14 ± 3.03	0.74 ± 0.58	31.98 ± 2.67	0.16 ± 0.16	41.72 ± 11.08	43.08 ± 11.90
12 (4)	76.86 ± 6.51	0.17 ± 0.16	22.58 ± 6.31	0.40 ± 0.68	27.80 ± 6.28	38.92 ± 24.15
13 (3)	64.70 ± 9.39	2.59 ± 0.57	31.82 ± 9.07	0.89 ± 0.69	50.58 ± 5.30	58.27 ± 2.76
14 (4)	78.73 ± 3.55	0.98 ± 0.80	20.08 ± 4.01	0.24 ± 0.91	38.26 ± 11.06	43.10 ± 13.15

*MFl*, mean fluorescence; *H*_*2*_*O*_*2*_, hydrogen peroxide

**Table 5:** Stallion sperm superoxide status (n = 14 stallions, 58 ejaculates)

Stallion (ejac no.)	live Superoxide negative	Live Superoxide positive	Dead Superoxide negative	Dead Superoxide positive	MFI Live Superoxide positive	MFI Dead Superoxide positive
1 (3)	47.97 ± 4.77	15.49 ± 16.32	2.47 ± 0.86	34.08 ± 10.69	334.54 ± 106.99	2008.30 ± 654.37
2 (1)	65.27	7.20	1.41	26.11	191.33	1771.63
3 (5)	60.60 ± 6.13	9.68 ± 1.80	1.74 ± 0.70	27.99 ± 4.31	144.22 ± 48.60	1346.07 ± 391.91
4 (9)	56.49 ± 6.98	10.30 ± 5.78	3.04 ± 1.18	30.17 ± 7.68	212.98 ± 85.57	1439.26 ± 496.87
5 (5)	46.73 ± 13.78	6.10 ± 2.59	2.73 ± 1.75	44.44 ± 11.53	204.24 ± 125.09	1747.08 ± 334.18
6 (3)	59.29 ± 10.73	3.13 ± 0.39	5.19 ± 1.73	32.38 ± 9.54	110.37 ± 29.47	1116.74 ± 119.45
7 (4)	51.13 ± 13.08	15.87 ± 7.80	2.39 ± 1.19	30.61 ± 12.89	177.38 ± 45.16	919.23 ± 176.88
8 (4)	65.78 ± 19.53	15.90 ± 17.98	0.94 ± 0.65	17.38 ± 3.54	244.30 ± 207.15	1414.93 ± 314.62
9 (4)	47.81 ± 13.25	17.87 ± 7.24	4.75 ± 6.68	29.57 ± 14.58	272.23 ± 86.46	1243.29 ± 453.79
10 (5)	33.04 ± 9.49	6.22 ± 1.53	1.70 ± 0.78	59.04 ± 9.51	182.74 ± 92.24	1280.85 ± 462.56
11 (4)	53.92 ± 8.69	13.98 ± 10.52	1.71 ± 1.01	30.39 ± 1.95	245.62 ± 120.76	1299.56 ± 153.95
12 (4)	68.63 ± 7.44	8.37 ± 1.02	1.31 ± 0.72	21.70 ± 6.12	186.39 ± 23.68	1308.39 ± 141.47
13 (3)	61.66 ± 12.50	5.63 ± 2.88	1.58 ± 0.26	31.12 ± 9.85	161.86 ± 8.01	1074.88 ± 74.01
14 (4)	74.99 ± 4.50	4.68 ± 1.31	1.93 ± 0.66	18.41 ± 3.94	108.98 ± 11.52	1087.58 ± 144.14

*MFl*, mean fluorescence

**Fig. 2:**
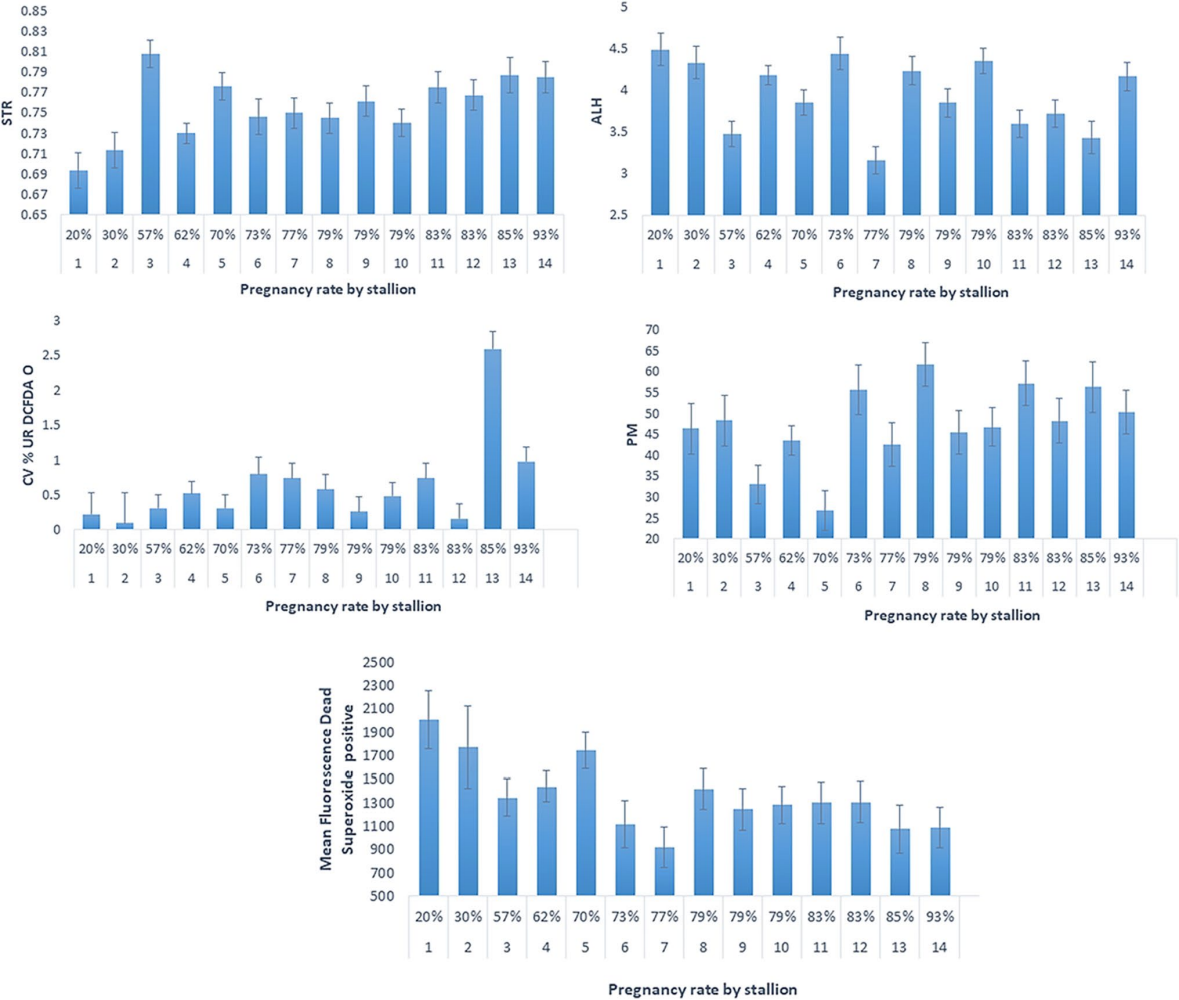
Association between pregnancy rate and straightness, amplitude of lateral head displacement, live hydrogen peroxide positive spermatozoa, progressive motility, and dead superoxide positive spermatozoa in stallion semen (n = 14). Note: STR = Straightness, ALH = Amplitude of lateral head displacement, CV% UR DCFDA = live, hydrogen peroxide positive, PM = progressive motility

Correlations between the different measured parameters and fertility are provided in Supplementary Table 1. Significant correlations were found between pregnancy rate and five parameters of sperm quality, as shown for individual stallions (Fig. 2) and summarized in Table 6. Positive correlations were found between pregnancy rate and STR (r = 0.43, p ≤ 0.001), and pregnancy rate and production of live spermatozoa hydrogen peroxide (r = 0.32, p ≤ 0.05). There were negative correlations between pregnancy rate and amplitude of lateral head displacement (r = -0.26, p ≤ 0.05), and between pregnancy rate and dead spermatozoa positive for superoxide (r = -0.46, p < 0.001).

**Table 6:** Significant correlations (r, p value) between sperm quality and pregnancy rate

		STR	ALH	Live H_2_O_2_ + spermatozoa	Mean fluorescence of Dead SO^.^ + spermatozoa	PM
Pregnancy rate	**r**	0.43	-0.26	0.23	-0.46	0.22
	**p**	0.001	0.04	0.09	0.0004	0.09

*STR*, straightness; *ALH*, amplitude of lateral head displacement; *H*_*2*_*O*_*2*_, hydrogen peroxide; *SO*^*.*^, superoxide; *PM*, progressive motility

The five parameters showing a p-value below 0.1 were further tested for their sensitivity, specificity and accuracy for identification of stallions with fertility resulting in ≥ 70% pregnancy rate or fertility resulting in < 70% pregnancy rate. The results (Table 7) show, in general, a high sensitivity but a moderate specificity and accuracy for these predictions.

**Table 7:** Sensitivity, specificity and accuracy of progressive motility, straightness, amplitude of lateral head displacement, live hydrogen peroxide production and mean fluorescence of dead superoxide positive population for identification of stallions with ≥ 70% pregnancy rate or < 70% pregnancy rate

Variable	Threshold	Sensitivity (%)	Specificity (%)	Accuracy (%)
Progressive motility	57.5	93.33	42.22	55
Straightness	0.77	77.78	42.42	58.3
Amplitude of lateral head displacement	< 4.37	71.11	46.67	65
Living hydrogen peroxide positive	0.72	85.71	39.13	50
Mean fluorescence dead superoxide positive	< 1545.59	72.34	53.85	68.3

**Fig. 3:**
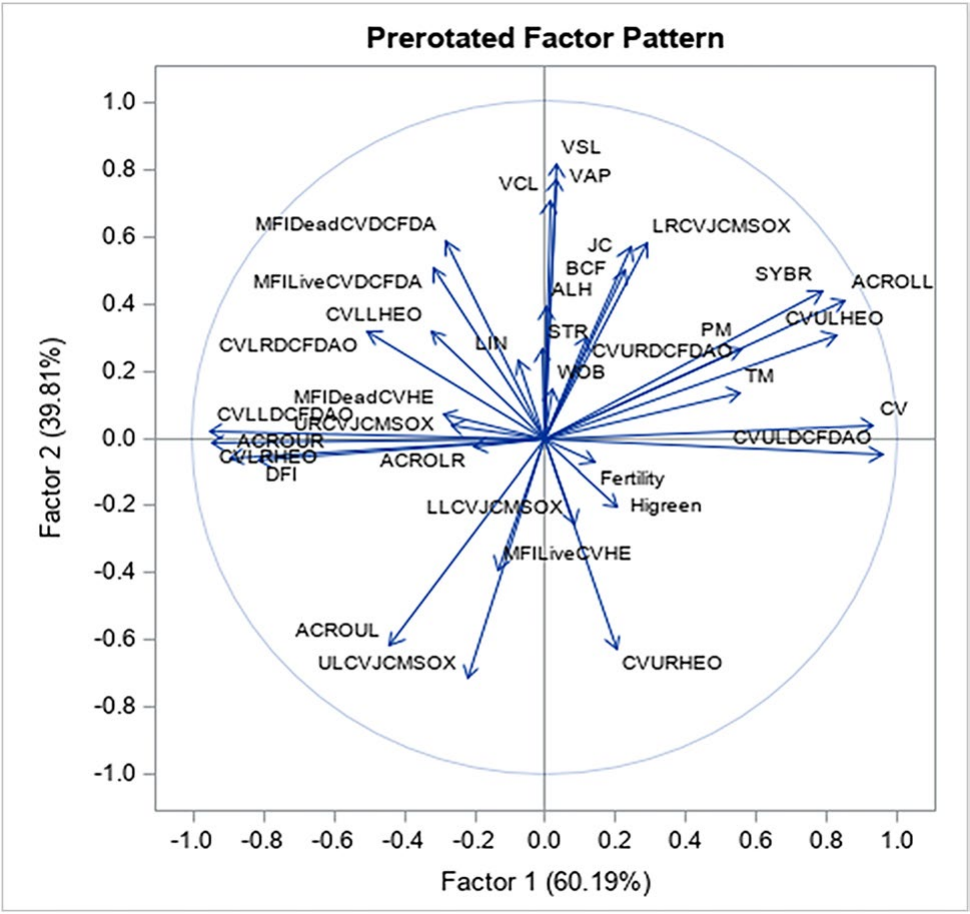
Principal component (PC) analysis of various stallion sperm characteristics associated with pregnancy rate, showing first (X-axis) and second (Y-axis) principal components. The first PC is mainly influenced by motility, membrane integrity, DNA fragmentation, and reactive oxygen species production, while the second is mainly influenced by sperm kinematics and reactive oxygen species production. Notes: PM = progressive motility, TM = Total motility, ACROLR = live acrosome reacted, URCVJCMSOX = high MMP high superoxide, MFI Dead CVHE = mean fluorescence dead, CVLLHEO = dead superoxide negative, CVLRDCFDAO = dead hydrogen peroxide positive, DFI = DNA fragmentation index (%), ACROUR = dead acrosome reacted, CVLRHEO = dead superoxide positive, CVLLDCFDAO = dead hydrogen peroxide negative, CVULDCFDAO = live hydrogen peroxide positive, CV = calcein violet, ACROLL = live non-acrosome reacted, CVULHEO = live superoxide negative; VCL = curvilinear velocity; VSL = straight line velocity; AVP = average path velocity; STR = straightness, LIN = linearity, WOB = wobble; ALH = amplitude of lateral head displacement; BCF = beat cross frequency, MFIDeadCVDCFDA = mean fluorescence hydrogen peroxide positive, LRCVJCMSOX = High mitochondrial membrane potential low superoxide, LLCVJCMSOX = low mitochondrial membrane potential low superoxide, ACROUL = dead non-acrosome reacted, ULCVJCMSOX = low mitochondrial membrane potential high superoxide, MFILiveCVHE = mean fluorescence live superoxide positive, MFILiveCVDCFDA = mean fluorescence live hydrogen peroxide positive, CVURHEO = live superoxide positive, CVURDCFDAO = live hydrogen peroxide positive, Higreen = high DNA staining, JC = high mitochondrial membrane potential

In a principal component analysis (Fig. 3 and Table 8), the first component reflects variables pertaining to viability and ROS production, whereas the second component is primarily shaped by kinematics. Specifically, the first principal component underscores motility, membrane integrity, DNA fragmentation, and reactive oxygen species production, while the second component is characterized by sperm kinematics and ROS production.

**Table 8:** Results of the principal component analysis based on sperm functional attributes in stallions with different fertility ratings

Component 1		Component 2	
Parameter	Loading factor^1^	Parameter	Loading factor^1^
CVULDCFDAO	0.99657	VSL	0.99770
CV	0.99769	VAP	0.99792
ACROLL	0.72962	VCL	0.99962
CVULHEO	0.82360	MFIDeadCVDCFDA	0.73262
SYBR	0.66731	LRCVJCMSOX	0.71766
PM	0.73036	JC	0.77959
TM	0.91488	MFILiveCVDCFDA	0.60251
Fertility	0.70678	BCF	0.75228
ACROLR	-0.97536	ALH	1.00000
URCVJCMSOX	-0.97059	CVURDCFDAO	0.81461
MFIDeadCVHE	-0.91975	STR	0.99913
CVLLHEO	-0.36282	LIN	0.86104
CVLRDCFDAO	-0.60540	WOB	0.97568
DFI	-0.98989	Higreen	-0.35605
ACROUR	-0.99427	LLCVJCMSOX	-0.85374
CVLRHEO	-1.00000	MFILiveCVHE	-0.84845
CVLLDCFDAO	-0.99952	ACROUL	-0.53908
		CVURHEO	-0.86291
		ULCVJCMSOX	-0.87021

^1^The loading factor represents the highest association between a given sperm functional attribute and the corresponding principal component. *PM*, progressive motility; *TM*, Total motility; *ACROLR*, live acrosome reacted; *URCVJCMSOX*, high MMP high superoxide; *MFI Dead CVHE*, mean fluorescence dead; *CVLLHEO*, dead superoxide negative; *CVLRDCFDAO*, dead hydrogen peroxide positive; *DFI*, DNA fragmentation index (%); *ACROUR*, dead acrosome reacted; *CVLRHEO*, dead superoxide positive, *CVLLDCFDAO*, dead hydrogen peroxide negative; *CVULDCFDAO*, live hydrogen peroxide positive; *CV*, calcein violet; *ACROLL*, live non-acrosome reacted; *CVULHEO*, live superoxide negative; *VCL*, curvilinear velocity; *VSL*, straight line velocity; *AVP*, average path velocity; *STR*, straightness; *LIN*, linearity; *WOB*; wobble; *ALH*; amplitude of lateral head displacement; *BCF*, beat cross frequency; *MFIDeadCVDCFDA*, mean fluorescence hydrogen peroxide positive; *LRCVJCMSOX*, High mitochondrial membrane potential low superoxide; *LLCVJCMSOX*, low mitochondrial membrane potential low superoxide; *ACROUL*, dead non-acrosome reacted; *ULCVJCMSOX*, low mitochondrial membrane potential high superoxide; *MFILiveCVHE*, mean fluorescence live superoxide positive; *MFILiveCVDCFDA*, mean fluorescence live hydrogen peroxide positive; *CVURHEO*, live superoxide positive; *CVURDCFDAO*, live hydrogen peroxide positive; *Higreen*, high DNA staining; *JC,* high mitochondrial membrane potential

## Discussion

Here, we report a study aimed at a better prediction of stallion fertility, wherein vitro sperm quality parameters could be used to identify stallions with a pregnancy rate above 70%. The interest in predicting stallion fertility is obvious, but complicated by the fact that fertility is a multifactorial phenomenon, and is dependent on insemination factors as well as mare and stallion factors, in addition to the sperm factors that can be studied in the laboratory. Motility is commonly used to decide if semen can be used for AI despite its poor predictive value for fertility (Morrell et al. 2017). Even if a spermatozoon is motile, it may have morphologic abnormalities or damaged chromatin, which affect pregnancy rates (Morrell et al. 2008). Thus, the evaluation of other parameters in addition to motility is important (Love et al., 2002).

In the present study, the parameters that were most helpful in identifying the stallions with a pregnancy rate above 70% were kinematics and ROS production. Of the kinematic variables, STR and PM both showed a positive correlation to pregnancy rate while ALH showed a negative correlation. Correlations between both TM and PM with fertility was observed in a previous study from our group (Morrell et al 2014), while Suliman et al (2020) found differences in TM, PM, VCL and VAP between fertile and sub-fertile stallions. In contrast, Vidament (2005) found an association between rapid motility and fertility, and between VAP and fertility. They did not find correlations between STR, PM or ALH and stallion fertility. The different outcomes of these studies may be due to different CASA instruments with different algorithms and different settings.

Unexpectedly, in our study there was a positive correlation between living spermatozoa producing hydrogen peroxide and pregnancy rate, and a negative correlation between mean fluorescence of dead superoxide positive spermatozoa and pregnancy rate. Gibb et al. (2014) observed the apparent paradox of an association between fertility and oxidative stress in fresh semen samples. The same principle that they used to explain their results could apply to the present experiment, namely that if the spermatozoa from more fertile samples were metabolizing at a faster rate than those in less fertile samples, they could be generating more ROS. In contrast, another study, albeit with different fluorochromes and imaging flow cytometry, showed a relationship between ROS generation and some types of abnormal sperm morphology (Bulkeley et al. 2023), providing concrete evidence that ROS generation is linked to certain morphological defects. Since morphological evaluation was not carried out on the samples in the present study, we do not know if our samples would have had the same morphological defects as in the study by Bulkeley et al. (2023).

Aurich et al. (1997) summarized the events leading to a reduction in fertility in cooled stallion semen, which involve peroxidation of sperm membrane lipids leading to decreased membrane integrity and impairment of function, together with decreased motility. Reactive oxygen species are believed to contribute to this lipid peroxidation but physiological concentrations of these substances are required for hyperactivation, capacitation and the acrosome reaction (Izadpanah et al. 2015), without which the sperm would be unable to fertilise an oocyte. Although ROS are metabolic byproducts, and as such are indicators that the sperm are able to function, there may be a mismatch between ROS production and their inactivation by antioxidants in stored semen samples, leading to increased ROS concentrations and thus to sperm membrane damage. However, our results did not show a negative association between ROS production and membrane integrity or fertility, suggesting that the semen extenders were able to balance ROS-production and inactivation in these samples.

In previous studies from our group, %DFI was one of the most useful variables for predicting stallion fertility (Morrell et al. 2014; Al-Kass et al. 2023). Surprisingly, in our present study, the %DFI variable was not significantly correlated with pregnancy rate, possibly due to the small number of stallions included. However, inferences made from fertility trials are tenuous if the sample sizes are small (Amann 2005). Moreover, imprecision could occur due to the use of “per season” pregnancy rates instead of per cycle pregnancy rates, and the high variability in the number of mares inseminated per stallion (15 to 320).

Apart from %DFI, the SCSA also provide an evaluation of high DNA stainability sperm (Higreen). According to Evenson (2013), high DNA stainability is indicative of retained histones in the nucleosomes of the DNA, which are associated with subfertility in humans. However, there was no association between Higreen and pregnancy rate for the samples analysed here.

As mentioned previously, the ability of sperm to undergo the acrosome reaction is a crucial step in fertilization, without which the sperm will not be able to bind to the zona pellucida (Gadella et al., 2001). However, the sperm must only acrosome-react in the presence of the oocyte and do not survive long in the reacted state. Therefore, the presence of a high proportion of acrosome-reacted sperm in the ejaculate before insemination could result in an inability to achieve fertilization. Although the proportions of live acrosome-intact sperm varied between 36 and 82% in this study, there was no indication that this characteristic could be used as a biomarker of fertility. However, just because the sperm have intact acrosomes does not mean that they will be able to acrosome-react at the appropriate time. In a previous study, a positive correlation was observed between fertility and the proportion of acrosome-reacted sperm after stimulation (Tello-Mora et al. 2018). In our study, the acrosome status was measured only in unstimulated samples. Furthermore, Ortiz et al. (2021) reported that agglutination of sperm might be a problem during flow cytometric analysis, which was a problem in our samples. 

In previous studies, high MMP was observed to be associated with sperm functionality, at least in human and bovine semen samples (Sousa et al. 2011) but to our knowledge, few studies have evaluated MMP and ROS simultaneously. In a previous study, we reported that such a simultaneous measurement in stallion sperm provided useful additional information (Johannisson et al. 2018). Our hypothesis was that mitochondrial ROS (superoxide) production could provide an indication of how metabolically active the sperm are and thus provide an indication of their potential fertility. Previously, Akbarinejad et al. (2020) found that MMP and superoxide production were correlated in stallion sperm. In the present study, MFL for dead SO +  was negatively correlated with fertility. The intensity of the fluorescence (MFl) provides an indication of how much of the particular parameter is present. Thus, we suggest that this measurement gives additional information about the sperm sample that could be useful in predicting its fertility potential. Since SO in the sperm sample could be readily converted to H_2_O_2_, we speculate that high concentrations increase the risk of damaging viable sperm during storage. The high correlation observed in this study is a novel finding requiring further investigation.

Petrunkina & Harrison (2011) pointed out the limitations of performing individual assays for different aspects of sperm quality, recommending instead that simultaneous analysis should be carried out by flow cytometry. However, several of the fluorophores used for sperm function assays emit fluorescence in the green or red channels, thus limiting the number of assays that can be run simultaneously. In contrast, calcein violet fluorescence is a blue fluorochrome, allowing it to be combined with other fluorophores for analysis, to facilitate identification of live cells (Egyptien et al. 2023). Simultaneous evaluation of other sperm characteristics is warranted in the search for biomarkers of stallion sperm fertility, and could be helpful in determining what could be incorporated in a fertility prediction model.

Fertility is a multifaceted phenomenon, involving factors related to sperm quality and the mare, as well as extraneous factors, such as semen handling protocols (Morrell et al. 2014). Therefore, the fertility potential of a sperm sample does not rely on a single characteristic, which is why predictions based on one parameter have poor predictive value. Fertility prediction in the horse is made more complicated by the fact that each ejaculate is used for AI in only a few mares, and the cumulative statistics over the whole of the breeding season may involve relatively few females per stallion (Amann 2005). Adding more parameters to the model increases its predictive value (Pena 2018; Johannisson et al. 2018), but the relevant combinations of factors relating to fertility need to be identified and combined. Moreover the problem of spectral overlap has to be considered (Petrunkina & Harrison 2011; Quirino et al. 2022). The present study contributed to this knowledge by investigating the use of new combinations of fluorophores incorporating calcein violet, as well as by identifying MFl as a potential biomarker. However, identifying more candidates to add to the model would improve its predictive value.

## Conclusion

Motility, sperm kinematics and aspects of reactive oxygen production were all associated with per season pregnancy rate; a combination of these factors could be used as a biomarker of fertility when assessing ejaculates. Flow-cytometric analysis with appropriate probes thus offers considerable promise for the prediction of stallion fertility.

### Supplementary Information

Below is the link to the electronic supplementary material.

Supplementary file1 (XLSX 23 KB)

## Data Availability

All data supporting the findings of this study are available within the paper and its Supplementary Information.
